# L1 Mandarin children’s acquisition of the “One + Classifier” structure: empirical evidence and insights from special language domain theory

**DOI:** 10.3389/fpsyg.2026.1763771

**Published:** 2026-07-06

**Authors:** Nuoyi Lin, Mingjie Zhou

**Affiliations:** 1 Department of Chinese Language and Literature, University of Macau, Taipa, Macau SAR, China; 2 Department of Chinese Language and Literature, Huazhong University of Science and Technology, Wuhan, China

**Keywords:** “One + Classifier” structure, CHILDES-TCCM, error analysis, L1 Mandarin child language acquisition, special language domain theory

## Abstract

**Introduction:**

This study examines early Mandarin acquisition of the *yi* “one” + classifier construction in monolingual Taiwanese Mandarin-speaking children and asks whether numeral-classifier development is holistic or staged.

**Methods:**

Using the CHILDES-TCCM corpus, we analyzed 35,864 child utterances from 126 transcript files produced by ten children. We isolated 554 screened yi-target records, compared them with a matched caregiver-input baseline, and retained compressed adult headline materials as a qualitative contrast.

**Results:**

Overt *yi* + classifier forms overwhelmingly dominated the child data: 543 of the 554 screened records were target-like. Only four clear omissions and two classifier-form errors were identified, and five additional tokens were excluded as ambiguous. Clear omissions were rare but concentrated in the 2;00–3;00 age range and distributed across three children. Individual classifiers dominated throughout, and child output was even more strongly skewed toward individual classifiers than caregiver input. The classifier ge functioned as an early broad-use form, but post hoc review showed that only a small subset of *yi* ge + N tokens constituted clear overgeneralization.

**Discussion:**

The comparison with headline data is retained as a qualitative contrast. It does not motivate a shared derivational mechanism, but shows that under strong discourse pressure, surface Num + N strings remain interpretable in Mandarin. Overall, the findings support a staged and non-holistic view of numeral-classifier development while treating the evidence for omission cautiously because of the small number of clear omission tokens.

## 1 Introduction

Numerals and classifiers are central to Mandarin acquisition, and a substantial body of research has addressed their learning. In the grammar of Mandarin, numerals and classifiers are distinct grammatical categories, yet their separate use is tightly constrained. As Li (2000) notes, classifiers support numerals in counting and ordering; accordingly, Mandarin typically expresses quantity through a numeral–classifier structure in which the two elements jointly carry the relevant semantic load. Since ordinary nouns in Mandarin cannot normally be modified directly by numerals, the insertion of a classifier is grammatically required, making the numeral–classifier structure a basic building block of Mandarin noun phrases.

The classifier system in Mandarin is large and formally complex, with different classifiers serving different functions and selectional patterns. Sheng (2003) emphasizes that classifiers not only mark quantity but also participate in rhetorical extensions and structural variation. In A *Grammar of Spoken Chinese*, Chao (1968) classifies classifiers into nine major types and details their pairing restrictions and syntactic behavior. In Chao’s system, each noun typically has one or more preferred classifiers, alongside the general classifier *ge*. The general classifier ge shows unusually wide compatibility across noun classes, and *yi* ‘one’ combines productively with almost all classifiers (see Table 1).

**Table 1:** Chao’s (1968) nine categories of classifiers in *Grammar of Spoken Chinese.*

Classifier category	Occurrence with numerals greater than 1	Examples of classifiers	Occurrence with *de*	Examples of nouns
Individual classifier	✓	*ge 个/wei 位/zhi 只/duo 朵/jia 家/liang 辆*	*(no *de* is inserted between the classifier and the noun)	*ren*人
Classifier occurring in V–O constructions	✓	*ju*句/*shou*手/*jie*届/*pan*盘	()	*hua*话/*haozi*好字/*qi* 棋
Collective classifier	✓	*hang*行/*dui*对/*shuang*双	()	*zi*字/*jiezhi*戒指/*yanjing*眼睛
Partitive classifier	✓	dui堆/fen份/kuai块	()	*tu*土/*liwu*礼物/*shitou*石头
Container classifier	✓	*guo*锅/*xiang*箱/*ping*瓶	()	*mian*面/*shu*书/*shui*水
Temporary classifier	*(cannot occur with numerals other than *one*)	*di*地/*lian*脸/*shen*身	√	*dongxi*东西/*han*汗/*xue*雪
Standard classifier	✓	*chi*尺/*mu*亩	√	*bu*布/*di*地
Quasi-inherent/autonomous classifier	✓	*heng*横/*guo*国/*ke*课	*	*
Verbal classifier	✓	*tang*趟/*bian*遍/*quan*圈	*	*

“()” means *de* is normally not permitted between the classifier and the noun, except in very unusual or marked contexts. * indicates that the corresponding combination is not applicable or not permitted in ordinary Mandarin.

Despite this descriptive background, existing research has rarely examined the numeral–classifier structure as an integrated whole, even though numerals and classifiers have only limited independent use in ordinary Mandarin (Li, 2000). Previous studies have also focused mainly on children above the age of three, leaving a gap in our understanding of how children between one and three acquire this structure in early spontaneous speech. Corpus data from CHILDES-TCCM (MacWhinney, 2000) show that during this period the overwhelming majority of numeral–classifier tokens involve *yi* ‘one’. Because *yi* is the earliest, most frequent, and most semantically accessible numeral in the corpus, the present study focuses on the *yi* + classifier structure.

From a developmental perspective, numerals and classifiers place partially different demands on the learner. Carey (2004) argues that young children first acquire number words through rote routines and only gradually connect them to exact quantity. Sang (2016) likewise shows that numeral comprehension is sensitive to the child’s grasp of numerical meaning and to the processing demands introduced by nominal material. Research on classifier acquisition has repeatedly shown that not all classifier types emerge at the same time. Individual classifiers, especially *ge*, are reported to appear early, while more specific or less frequent types emerge later (Ying et al., 1983; Ding, 1999; Peng and Chen, 2016; Fang, 1985). More recent work also suggests that children’s development of numerals and classifiers is related rather than independent, with *ge* often functioning as an early placeholder before finer distinctions are stabilized (Chen and Her, 2025).

These acquisition facts also bear on lexical-semantic development. Classifier choice is not determined by phonological form alone. To select an appropriate classifier, the child must construe the referent as an individuated object, a bounded quantity, a flat or elongated shape, a living creature, a portion, or a measurable unit. As Bloom (2000) argues more generally, word learning depends on the interaction of conceptual, social, and linguistic capacities rather than on simple surface co-occurrence. In the classifier domain, this means that acquisition requires not only memorizing a form, but also learning what kind of entity, portion, event, or measure that form picks out. This helps explain why classifier development may remain uneven even after numeral yi has already become available.

Within this broader acquisition picture, Universal Grammar (UG) provides the article’s theoretical backdrop rather than a direct explanation for any single error type. On a standard generative view (Chomsky, 1988), acquisition begins from a biologically given initial state and proceeds through intermediate grammars under the pressure of input. What matters here is a narrower consequence of that view: If child grammars are genuine grammatical systems, then early non-target productions should be limited and structured, not freely random. For Mandarin numeral–classifier structures, this expectation directs attention to whether errors show a consistent asymmetry—such as retention of *yi* with occasional classifier omission—instead of unrestricted loss, classifier-only forms, or disorder within the nominal structure.

Alongside this acquisition perspective, the article also draws on Xu and Qin’s (2015) notion of “Special Language Domain” (SLD). Xu and Qin use this term for genres such as headlines, slogans, poetry, and network discourse in which speakers may, under identifiable communicative pressures, depart from ordinary particular grammar while remaining interpretable and still operating within broader linguistic constraints. Later work on SLD further emphasizes that such innovation is not unconstrained: it is bounded by the larger grammar and tends to occupy licit gaps or edge positions in the system rather than creating arbitrary forms (Dong and Huang, 2019). In the present study, the relevance of SLD is narrower than in the original submission. One recurrent property of headline-type SLD is compression under information-management pressure, where overt material may be reduced while the intended message remains recoverable (Yao and Xu, 2021). This does not explain children’s omissions. Its value is contrastive: it helps distinguish questions about what adult Mandarin can tolerate on the surface under extreme compression from questions about why children produce occasional Num + N forms during development.

On this basis, the article addresses three questions: First, in the high-frequency *yi* + classifier structure, do children acquire numeral and classifier as a tightly integrated structure from the outset, or do they pass through a stage in which the numeral is stabilized earlier than the classifier? Second, how do classifier types and the productivity of the *yi* + classifier structure change across the sampled age range? Third, how should rare child Num + N omissions be interpreted in relation to caregiver input and to surface compression in Special Language Domain materials?

These considerations motivate three working hypotheses: (I) if the classifier-related part of the structure is not yet fully stabilized, children may occasionally produce numeral + noun forms while still preserving the numeral *yi*. (II) Classifier development is expected to be uneven across semantic types, with broad individual classifiers emerging earlier than more specialized forms. (III) If adult compressed genres also permit classifier-less Num + N strings under strong discourse pressure, these adult materials may help clarify the range of surface compression that Mandarin tolerates, even if they do not explain children’s acquisition directly.

Taken together, these considerations lead to several empirical expectations. Overt *yi* + classifier forms should dominate child speech, but rare non-target forms should preferentially take the shape of classifier omission rather than numeral omission. The distribution of classifier types should broaden with age, while ge may initially function as a general or placeholder form before more specific pairings are stabilized. Finally, if headline-like Num + N strings are used as qualitative comparison data, their value should lie in showing that surface compression of this kind is not wholly alien to Mandarin, not in claiming a shared derivational mechanism with child grammar.

## 2 Methods

### 2.1 Participants and corpus sources

The child data come from the TCCM subcorpus (Cheung and Chang, 2007) in the CHILDES system (MacWhinney, 2000), which documents naturalistic interactions of Mandarin-speaking children in Taiwan. Ten monolingual children were selected. Most observations fall between 1;05 and 3;11, although the Chw recordings contribute a small number of tokens up to 4;03 because this child has relatively sparse data before that point. The raw corpus sample used here contains 126 files, 35,864 child utterances, and 65,342 matched adult utterances.

Table 2 summarizes the participant overview and the size of the matched input–output sample. In addition to the raw file and utterance counts, it reports the number of pre-screened child *yi*-target records and the number of caregiver *yi* + classifier records retained for input comparison.

**Table 2:** Participant overview and matched input–output corpus size.

Child	Age range	Files	Raw child utterances	Raw adult utterances	Child *yi*-target records	Caregiver *yi* + CL records
Cheng	3;01–3;11	11	3,630	5,448	78	166
Chou	2;01–3;04	16	5,286	9,222	114	265
Chw	3;06–4;03	9	2,686	3,719	33	122
Jc	2;02–3;05	14	5,256	8,898	55	414
Pan	1;07–3;09	18	3,586	8,071	21	237
Wang	2;05–3;04	12	3,194	4,481	113	154
Wu	1;07–2;10	12	2,850	8,878	51	258
Wuys	2;07–3;10	10	1,904	5,771	55	287
Xu	1;06–2;05	11	2,721	4,522	9	145
Yang	1;05–2;09	13	2,751	7,332	25	391

The caregiver-input baseline was drawn from the same interactional files as the child records. Because caregiver speech in parent–child interaction often contains immediate echoes of the child’s preceding utterance, the input counts were de-duplicated before analysis. Adult repetitions of the child’s form, minimal recasts, and corrective prompts were excluded from the independent input totals. Thus, after a child produced *yi ben*, an immediate adult continuation such as *yi ben ya* was not counted as a separate input token; likewise, after a non-target child form such as *yi ba chuan*, an adult prompt like *yi shenme chuan*? *yi sou chuan* was treated as corrective feedback rather than as an independent input occurrence. The purpose of this step was to prevent caregiver counts from being inflated by local repetition or repair exchanges and to make the input baseline closer to the forms children actually heard as independent caregiver productions.

Special Language Domain material was collected separately as qualitative comparison data. Following Xu and Qin (2015), SLD is understood broadly to include headlines, slogans, poetry, lyrics, and related compressed genres. The present study uses mainly headline-type materials, since headline language has been repeatedly identified in the SLD literature as a particularly clear site of information compression and overt surface innovation (Xu and Qin, 2015; Yao and Xu, 2021). In strings such as *yi gongjiao siji* “one bus driver” or *yi jianshenfang* “one gym”, fuller Mandarin would ordinarily require a human or establishment/location classifier such as *ming/wei* and *jia/zuo/suo*, depending on the noun. For the qualitative comparison reported here, a small set of headline-type examples was reviewed and six representative cases are presented below; these materials were not entered into inferential statistics, but were used only to characterize bounded adult Num + N compression in a genre where reduction is deliberate and communicatively motivated.

### 2.2 Retrieval and coding of *yi*-target records

Using CLAN, *yi* “one” was used as the retrieval term. Each candidate token was examined in a three-turn local discourse window consisting of the target utterance plus the immediately preceding and following turns. The purpose of this step was to determine whether *yi* was genuinely count-denoting, whether the relevant noun phrase belonged to an obligatory classifier context, and whether the child’s production was independent rather than imitative.

For the child data, only spontaneous *yi*-target records in obligatory classifier contexts were retained for main analysis. Records were excluded if *yi* was not count-denoting, if the form was a fixed expression, if the utterance was a direct imitation of preceding adult speech, or if the apparent reduction was better explained by discourse-licensed ellipsis. Thus forms such as *na yi* ‘take one’ or *zhe yi* “this one”, where the nominal content is recoverable from local discourse, were treated as ambiguous and excluded rather than counted as classifier omission. By contrast, target-like forms such as *yi zhi mayi* ‘one ant’ and clear omissions such as *yi qiuqiu* ‘one ball’ were retained.

Following Vihman (1996), the study adopts an emergence criterion rather than a mastery criterion. A *yi* + classifier form is treated as stably emerged when it occurs in at least three non-imitative, contextually appropriate tokens from independent interactional contexts. This criterion is used to identify stable appearance of a form, not to claim complete mastery of the classifier involved.

Classifiers were assigned to Chao’s classifier categories. Because reviewer comments raised the issue of *ge* overgeneralization, all overt *yi ge* + N tokens were also reviewed *post hoc*. Each token was classified as target-like *ge*, clear *ge* overgeneralization, marginal colloquial *ge*, or ambiguous. Judgments about the expected adult classifier were based on a triangulation of matched caregiver input, standard grammatical description, and contextual interpretation.

### 2.3 Statistical analysis

The statistical analyses were chosen to answer four focused questions. First, how stable are target-like yi + classifier forms relative to non-target forms overall and across age bands? Second, do clear omissions cluster in a particular developmental window despite their rarity? Third, do classifier-type distributions differ across age bands and between child output and caregiver input? Fourth, does richer matched input correspond to richer child output at the level of individual children or child-age cells? To address these questions, chi-square tests were used for distributional comparisons, Fisher’s exact test for sparse omission counts, Spearman correlations for matched input–output associations, and Wilson 95% confidence intervals for age-band omission rates. Because clear omissions are rare, all inferential results are interpreted conservatively.

## 3 Results

### 3.1 Emergence and distribution of *yi* + classifier forms

Analysis of the CHILDES-TCCM (MacWhinney, 2000) corpus shows that children’s overt *yi* + classifier forms expand with age and are strongly dominated by individual classifiers. Table 3 retains the token-level overview of stably emerged classifier forms across children and age bands. As in the original dataset, *yi ge* is by far the most frequent early form, followed by a small number of additional individual classifiers and a later expansion into verbal, partitive, collective, and other categories.

**Table 3:** Stably emerged *yi* + classifier forms in children aged 1–3.

Quantifier type	*yi* + classifier	Participants	1-year-old productions	2-year-old productions	3-year-old productions
Individual classifier	*yi ge一个*	Cheng			68
Individual classifier	*yi ge一个*	Chou		23	23
Individual classifier	*yi ge一个*	Chw			19
Individual classifier	*yi ge一个*	Jc		5	24
Individual classifier	*yi ge一个*	Pan		5	14
Individual classifier	*yi ge一个*	Xu	5		
Individual classifier	*yi ge一个*	Yang		16	
Individual classifier	*yi ge一个*	Wang		42	46
Individual classifier	*yi ge一个*	Wu	3	35	
Individual classifier	*yi ge一个*	Wuys		4	27
Individual classifier	*yi zhi一只*	Cheng			3
Individual classifier	*yi zhi一只*	Chou		6	
Individual classifier	*yi zhi一只*	Jc			4
Individual classifier	*yi zhi一只*	Yang		3	
Individual classifier	*yi zhi一只*	Wu		4	
Individual classifier	*yi zhi一只*	Wuys			14
Individual classifier	*yi ben一本*	Jc		3	3
Individual classifier	*yi zhang一张*	Chou		3	
Individual classifier	*yi zhang一张*	Jc		3	
Individual classifier	*yi tai一台*	Chou		3	
Individual classifier	*yi duo一朵*	Jc		3	
Individual classifier	*yi ba一把*	Chw			8
Individual classifier	*yi liang一辆*	Chou			3
Individual classifier	*yi zhi一支*	Wuys			3
Verbal classifier	*yi kou一口*	Chou		5	
Verbal classifier	*yi kou一口*	Wang			3
Verbal classifier	*yi ci一次*	Wang		3	
Verbal classifier	*yi ci一次*	Cheng			3

At 1;00–2;00, overt *yi* + classifier production is extremely limited and entirely dominated by individual classifiers. Between 2;00 and 3;00, both token counts and type counts increase sharply, and the inventory expands beyond individual classifiers. By 3;00–4;00, individual classifiers remain dominant, but the overall distribution is broader and more stable. This developmental picture is supported by the age-band summaries (see Table 4).

**Table 4:** Child *yi*-target records by age band: target-like and non-target forms.

Age band	Total *yi* records	Target-like	Clear omissions	Form errors	Ambiguous/excluded	Clear omission % (95% CI)
1;00–2;00	12	11	0	0	1	0.00 (0.00–24.25)
2;00–3;00	240	230	4	2	4	1.67 (0.65–4.21)
3;00–4;00	302	302	0	0	0	0.00 (0.00–1.26)

Across the entire screened child dataset, 543 of 554 *yi*-target records are target-like. Only four clear omissions and two classifier form errors were identified; five additional tokens were excluded as ambiguous. By age band, the clear omission rates are 0/12 in the 1;00–2;00 band (0.00%, Wilson 95% CI [0.00–24.25%]), 4/240 in the 2;00–3;00 band (1.67%, Wilson 95% CI [0.65–4.21%]), and 0/302 in the 3;00–4;00 band (0.00%, Wilson 95% CI [0.00–1.26%]). These intervals show both that omission is rare overall and that the apparent concentration in the middle band must be interpreted cautiously.

The four clear omissions are distributed across three children rather than being confined to a single idiosyncratic case: one token each from Chou and Wang, and two tokens from Wu. This distribution does not license strong claims about a universal stage, but it does support a cautious developmental interpretation rather than a purely accidental one.

### 3.2 Classifier categories in child output and caregiver input

Table 5 shows that individual classifiers (Mc) dominate both child output and caregiver input, but the skew is stronger in child speech. In the 1;00–2;00 band, child output is entirely limited to individual classifiers, whereas caregiver input already contains a broad range of non-individual forms, especially verbal classifiers (Mv) and partitive classifiers (Mp). By 2;00–3;00 and 3;00–4;00, children begin to produce non-individual categories, but the range of forms and classifier categories remains narrower than that of the input.

**Table 5:** Classifier-category distribution by age band in child output and caregiver input.

Age band	Source	Mc	Mv	Mp	Mo	Mg	Mq
1;00–2;00	Child output	11	0	0	0	0	0
1;00–2;00	Caregiver input	237	114	43	2	2	2
2;00–3;00	Child output	202	13	8	5	1	2
2;00–3;00	Caregiver input	686	355	133	4	10	14
3;00–4;00	Child output	276	15	8	0	2	1
3;00–4;00	Caregiver input	533	189	103	0	7	5

Mc = individual classifiers; Mv = verbal classifiers; Mp = partitive classifiers; Mo = container classifiers; Mg = collective classifiers; Mq = quasi-inherent/autonomous classifiers.

This asymmetry is also visible at the level of specific classifier forms. In caregiver input, some non-individual classifiers are highly frequent yet only weakly represented—or entirely unattested—in child output. Table 6 highlights this point by juxtaposing high-frequency caregiver forms with their child counterparts. The purpose is not merely to show that input is richer in the abstract, but to test whether children preferentially reproduce the very non-individual forms that are most available in their immediate input.

**Table 6:** Selected high-frequency classifier forms in caregiver input and child output.

Classifier form	Broad category	Child output count	Caregiver input count
*ge 个*	Mc	374	1,016
*xia下*	Mv	3	369
*dian点*	Mp	1	251
*ci 次*	Mv	12	92
*kou口*	Mv	13	91
*tiao跳*	Mv	0	24
*shou手*	Mv	0	23
*bian遍*	Mv	1	20
*kuai块*	Mp	0	18
*tian天*	Mq	3	17

For example, the verbal classifier *xia* occurs 369 times in caregiver input but only three times in child output, and the partitive classifier *dian* occurs 251 times in caregiver input but only 1 time in child output. By contrast, some forms such as *ci* and *kou* show better, though still limited, uptake. The comparison therefore bears directly on the input question: children are not simply reproducing whichever non-individual classifiers are most frequent around them. Input matters, but it does not map onto output in a simple one-to-one way.

The matched correlations point in the same direction. Input token counts and input type diversity do not show significant monotonic correlations with child output token counts or child classifier-type diversity, either at the whole-child level or across matched child-age cells. These null correlations should not be read as evidence that input is irrelevant, but they do indicate that input richness alone does not linearly determine child production.

Table 7 summarizes the inferential analyses together with effect sizes and confidence intervals where applicable. Because non-target counts are extremely sparse, the table focuses on the exact test for clear omission concentration rather than on an omnibus age-band test over all non-target forms; for the latter pattern, Table 4’s Wilson confidence intervals are more informative. The next two rows compare the distribution of classifier types in caregiver input and child output. The final four rows test whether richer matched input predicts richer output at the level of children or child-age cells. The overall pattern is asymmetrical: distributional differences are clear, but direct monotonic input–output correlations are not. In other words, the table is meant to distinguish between two different questions—whether input and output have the same distribution, and whether richer input predicts richer output—and the answers are not the same.

**Table 7:** Inferential tests used in the study and the questions they address.

Analysis	Test statistic	Effect size	95% CI	*p*
Clear omission: 2;00–3;00 vs. other ages combined	Fisher exact test	OR not estimated under sparse zero-cell counts	—	*p* = 0.033
Caregiver-input classifier types across age bands	χ^2^(10) = 20.27	Cramér’s V = 0.06	—	*p* = 0.027
Child output vs. caregiver input classifier-type distribution	χ^2^(5) = 193.16	Cramér’s V = 0.25	—	*p* < 0.001
Per child: input tokens vs. output tokens	Spearman ρ = 0.20	ρ = 0.20	[−0.49, 0.74]	*p* = 0.578
Per child: input type diversity vs. output type diversity	Spearman ρ = 0.09	ρ = 0.09	[−0.57, 0.68]	*p* = 0.800
Matched child-age cells: input tokens vs. output tokens	Spearman ρ = 0.33	ρ = 0.33	[−0.18, 0.70]	*p* = 0.201
Matched child-age cells: input type diversity vs. output type diversity	Spearman *ρ* = 0.13	ρ = 0.13	[−0.38, 0.57]	*p* = 0.624

### 3.3 Syntactic structures and usage environments of the *yi* + classifier phrase

At age one, children were already able to use yi + classifier to modify a noun, as in *yi zhi mayi* ‘one ant’. By age two, the structure also occurred in slightly more elaborate environments. These included numeral + classifier + adjective strings such as *wo nalai yi ge honghongde gei ni kan* ‘I will take a red one and show it to you’ (Pan, 2;8), and numeral + classifier + adjective + noun strings such as *yi ge yuanyuande yao* ‘one round medicine’ (Wu, 2;5). In usage terms, *yi* + classifier forms were used both in spontaneous descriptions and as replies to caregiver prompts asking for quantity.

(1) *CHI: 这边有一只小鸟。(Cheng, 3; 1)

zhè biān yǒu yì zhī xiǎo niǎo

here be one-CL bird

‘There is a little bird here.’

(2) *MOT: 有几只蚂蚁?

yǒu jǐ zhī mǎ yǐ

be how-many-CL ant

“How many ants are there?”

*CHI: 一只蚂蚁。(Wu, 1; 7).

yì zhī mǎ yǐ

one-CL ant

‘One ant.”

### 3.4 Non-target forms: omission, form error, and *ge*

Clear omissions are illustrated in (3)–(5). These examples preserve yi and the nominal meaning, but the classifier position is absent. Importantly, not all superficially reduced yi forms were counted as omissions. Example (6) was excluded from the core statistics because the noun is absent and the utterance is licensed by discourse ellipsis rather than by a straightforward Num + N reduction. The non-target set therefore reflects a deliberately conservative coding policy.

(3) 房工就是一(朵)花很漂亮的(Wang, 2; 8)

fáng gōng jiù shì yí (duǒ) huā hěn piàoliang de

fang gōng just be one-(CL) flower very pretty DE

“fáng gōng is like a flower, very pretty.”

(4) 妈妈 一 球球 + …(Wu, 2; 4)

māma yì qiúqiú

mama one ball

Intended: “Mama, one little ball”/”Mom, (I want) a ball.”

(5) 妈妈 我 那 一 人 在 那里? (Wu, 2; 6)

māma wǒ nà yì rén zài nàlǐ

mama I that one person be at there

Intended: “Mom, I, that one person is over there?”

(6) 拿一。(Wu, 2; 6)

ná yì

take one

Intended: “Take one.”/”(I) take one.”

A separate *post hoc* review was conducted for overt *yi ge* + N tokens in which a more specific classifier was plausibly available. Twelve such tokens were flagged. Of these, three were coded as clear overgeneralization (e.g., *yi ge ji* ‘one chicken’, *yi ge jiuhuche* ‘one ambulance’, *yi ge qiangbi* ‘one wall’), eight as marginal colloquial alternatives (e.g., *yi ge lüdeng* ‘one streetlamp’, *yi ge yezi* ‘one leaf’), and one as ambiguous. All remaining overt *ge* tokens were treated as target-like for the purposes of the present corpus description. This result reinforces the broader distributional finding that *ge* is frequent and broad in early speech, but that clear overgeneralization constitutes only a small subset of *ge* use. By contrast, classifier-form errors were even rarer: in one token, Xu produced *yi bian* for target *yi ben* ‘one volume/book’. Such cases were counted separately from omissions because the classifier slot was overtly filled, but with the wrong lexical item (see Table 8).

**Table 8:** Surface comparison between child omissions and compressed headline strings.

Child errors	News headlines	Structural feature
Mama wo na yi (ge) ren zai nali? “Mom, that one person is over there?”	liù-hào-xiàn yì nánzǐ tōu shǒujī “Line-6 one man steal cellphone”	Num + NP
Zhe yi (ge) (yusan) “This one (umbrella)”	Běijīng yì jiànshēnfáng tū guānmén “Beijing one gym suddenly close”	Num + NP
Developmentally conditioned non-target production	Intentional genre-driven compression	

### 3.5 Qualitative comparison with special language domain data

The adult comparison data come from headline materials drawn from what Xu and Qin (2015) term Special Language Domain, a broader set of compressed genres that also includes slogans, poetry, lyrics, and related forms. The present study uses only a small headline-based subset as qualitative comparison data. In these examples, a numeral appears directly before a human, institutional, or locational noun where ordinary Mandarin would normally insert a classifier such as *ming/wei* before human-denoting nouns or j*ia/zuo* before institutions and sites. The relevance of these materials lies not in explaining child acquisition, but in showing that classifier-less Num + N strings can remain interpretable in adult Mandarin under strong pressure for brevity and information density. For example:

Omission before noun phrases denoting humans:

(7) 江西一中学领导挪用剬款炒股

Jiāngxī yì zhōngxué lǐngdǎo nuóyòng gōngkuǎn chǎogǔ

Jiangxi one middle school leader embezzle public funds trade stocks

‘A leader of a middle school in Jiangxi embezzled public funds to trade stocks.’ (China Jiangxi Network, 2016)

(8) 澳门女子4次往返珠海追寻一剬交司机

Àomén nǚzǐ sì cì wǎngfǎn Zhūhǎi zhuīxún yí gōngjiāo sījī

Macau woman four-CL round trip Zhuhai seek one bus driver

‘A Macau woman made four trips to Zhuhai seeking a bus driver.’ (*Dayue Net*, 2016).

(9) 6号线一男子偷手机 路人集体帮忙追

liù-hào-xiàn yì nánzǐ tōu shǒujī lùrén jítǐ bāngmáng zhuī

Line-6 one man steal cellphone passerby collective help chase

‘On Line 6, a man stole a cellphone, and passers-by collectively helped chase him.’ (Guangming Picture, 2016)

Omission before noun phrases denoting locations:

(10) 北京一健身房突关门

Běijīng yì jiànshēnfáng tū guānmén

Beijing one gym suddenly close

‘A gym in Beijing suddenly closed down.’ (Beijing Morning Post, 2016)

(11) 美国俄克拉何马州一机场发生枪击事件

Měiguó Ékèlāhémǎ-zhōu yì jīchǎng fāshēng qiāngjī-shìjiàn

U. S. Oklahoma-State one airport occur shooting-incident

‘A shooting incident occurred at an airport in Oklahoma, USA.’ (People’s Daily, 2016)

(12) 天津一垃圾焚烧厂被指环评造假

Tiānjīn yì lājī fénshāo-chǎng bèi zhǐ huánpíng zàojiǎ

Tianjin one garbage incineration-plant PASS accuse environmental review falsify

“A garbage incineration plant in Tianjin was accused of falsifying its environmental assessment.” (China National Radio [CNR] Online, 2016)

## 4 Discussion

### 4.1 The hierarchy of classifier acquisition and semantic development

The corpus suggests a staged pattern in early classifier use. Individual classifiers are attested earliest and dominate throughout the sampled period; non-individual categories appear later and remain much rarer. This pattern accords with earlier studies that place broad individual classifiers, especially *ge*, at the beginning of classifier development (Ying et al., 1983; Ding, 1999; Fang, 1985; Peng and Chen, 2016). The developmental contrast is therefore not simply one of token count, but of semantic demand: early child speech relies heavily on forms that can be mapped onto highly concrete and readily individuated referents.

This developmental contrast is not only a matter of frequency. To choose a classifier, the child must attend to how the referent is construed: as an individuated object, a flat piece, a bounded portion, a containerful, an event count, and so on. In this sense classifier acquisition is tied to semantic development. Bloom (2000) argues more generally that word learning depends on the interplay of conceptual, social, and linguistic capacities rather than on simple pairing. In the classifier domain, this means that children are learning not only which form accompanies a noun, but what kind of entity or unit that noun is being construed as. The early dominance of individual classifiers is therefore consistent with the idea that concrete individuation is available earlier than more abstract or measure-based construals. Erbaugh’s (1986) observation that early classifier use tends to preserve concrete classifier–noun pairings fits this picture.

This does not, however, license a strong causal claim about cognitive load. The present corpus cannot distinguish whether the rarity of standard, temporary, and V–O-related classifiers reflects semantic abstraction, discourse rarity, limited production opportunities, or greater processing demands. What can be said is narrower: those later-emerging categories place heavier semantic demands on the child because they require finer construal of portions, measures, or event units. Independent work on children’s cognition shows that performance declines when processing and storage compete for limited resources and when concurrent cognitive demands rise (Chen et al., 2022; Schmid et al., 2007). These studies make a processing contribution plausible, but the present corpus does not test it directly. Any appeal to processing demand must therefore remain indirect and should be treated as one possible factor alongside semantic development and input distribution.

(13) *MOT: 请问这火腿怎么做啊?

qǐngwèn zhè huǒtuǐ zěnme zuò a

please ask this ham how make SFP

“May I ask how to cook this ham?”

*MOT: 很好吃耶!

hěn hǎochī ye

very tasty SFP

‘It tastes really good!’

*CHI: 切一片一片的!(Wang, 2; 11)

qiē yí piàn yí piàn de

cut one slice one slice DE

‘Cut it into slices!’/‘Cut it slice by slice!’

### 4.2 Non-holistic acquisition, UG, and competing explanations

The corpus supports a non-holistic account of *yi* + classifier development. The clearest asymmetry is narrow and position-specific: the rare non-target forms preserve *yi* while omitting the classifier. No corresponding cases were found in which a classifier was retained but *yi* was missing, and the child data do not show unrestricted disruption of noun-phrase order. This matters for the UG discussion because, on a continuity view of acquisition, intermediate grammars are expected to generate constrained innovations rather than arbitrary fragments.

This pattern can also be situated within a broader cross-linguistic literature on early weakening of grammatical material. In telegraphic speech and related early stages, children often omit function words and inflectional material before adult-like marking becomes stable (Brown, 1973). More specifically, determiner and article omission have been documented across several child languages, and the cross-linguistic pattern is not reducible to raw input frequency alone (Baauw et al., 2002; Guasti et al., 2008; de Lange, 2008). The present Mandarin pattern fits this broader cross-linguistic picture: classifier omission, like article omission, appears to involve a localized vulnerability in functional material rather than a direct reflection of raw input frequency. Related findings from language impairment are also relevant: children with SLI have been shown to omit obligatory relative markers in structures that require overt material in the CP domain (Schuele and Tolbert, 2001). These phenomena are not identical to Mandarin classifier omission, but they converge on a broader point: grammatical material may be selectively fragile in early or vulnerable systems even when lexical structure remains available. The Mandarin pattern observed here can therefore be placed within a wider family of functional-category weakness, with the classifier slot as the language-specific locus of vulnerability.

This observation does not prove a UG account, nor does it by itself eliminate frequency-based or processing-based explanations. It does, however, sharpen what those alternatives would need to explain. In the article-omission literature, Guasti et al. (2008) likewise argue that adult input frequency does not bear a systematic relation to child omission across languages. The present corpus shows a parallel mismatch: the matched caregiver baseline contains 2,488 overt *yi* + classifier records, overwhelmingly favoring the adult target pattern, yet clear omissions still surface in the child data. If child performance simply mirrored token frequency, such dense overt input should suppress Num + N forms much more strongly and should yield a closer lexical match between input and output. A pure processing-bottleneck account, if stated without structural constraints, would more naturally predict broader truncation or instability, potentially affecting *yi* itself or producing less position-specific reduction. What is actually observed is narrower: almost all records are target-like, and the rare non-target cases specifically involve omission of the classifier slot.

The claim is therefore modest. The present data are compatible with a structurally constrained account of early error and do not by themselves falsify usage-based or processing accounts. The narrower point is that frequency and processing alone do not straightforwardly predict why the vulnerability should fall so specifically on classifier realization.

### 4.3 Caregiver input, individual differences, and the general classifier *ge*

The caregiver baseline helps clarify both what children hear and what they do not straightforwardly reproduce. At the broad level, caregiver input is richer than child output in classifier-type distribution and lexical diversity, yet early child production remains more heavily concentrated in individual classifiers. The form-level mismatches discussed below should therefore be interpreted against this broader asymmetry between the distributional richness of the input and the narrower range of the child’s early uptake.

This mismatch becomes clearer at the level of specific forms. As Table 6 shows, several non-individual classifiers are highly frequent in caregiver input but remain weakly represented in child output. For example, *xia* occurs 369 times in caregiver input but only three times in child output, and *dian* occurs 251 times in caregiver input but only 1 time in child output. The issue is therefore not simply whether children hear non-individual classifiers, but how selectively they take them up in early production.

Variation among children is nonetheless substantial. Jc and Chou are particularly productive, while others remain more limited. Yet these individual differences are patterned rather than random: overt *yi* + classifier forms dominate across the sample, individual classifiers lead the distribution in every age band, and the small number of clear omissions is confined to a subset of children rather than spread evenly across the corpus. The present sample allows such differences to be described, but not exhaustively modeled.

The role of *g*e requires separate mention.

The general classifier *ge* occurs throughout the corpus and partly functions as an early broad-use form. This does not mean, however, that every *yi ge* + N token should be treated as an error. *Post hoc* review showed that some tokens are fully target-like, some are only marginally less preferred than a more specific classifier, and only a small subset qualify as clear overgeneralization. The developmental issue is therefore not the presence of *ge* itself, but children’s reliance on *ge* where finer lexical distinctions are not yet stable. For example:

(14) *EXA: J@s: eng 阿姨怎么喝?

āyí zěnme hē

aunt how drink

“Auntie, how do you drink (it)?”

*CHI:装一个咖啡。(Chou, 2; 4).

Zhuāng yí gè kāfēi

fill one CL coffee

“Pour (me) a coffee”/”Fill one coffee.”

(15) *MOT: 我看到一件好漂亮的衣服耶!

wǒ kàndào yí jiàn hǎo piàoliang de yīfú ye

I see one CL very pretty DE clothes SFP

“I saw a very beautiful piece of clothing!”

*CHI: 哪一个?(Wang, 3; 1).

nǎ yí gè

which one CL

“Which one?”

### 4.4 What the special language domain comparison contributes

The comparison with Special Language Domain data is intended primarily as a descriptive and contrastive comparison. It is not needed to show that child Num + N forms are systematic; that follows from the child corpus itself. Its added value lies elsewhere. Adult headline materials provide a contrast case in which surface reduction is intentional, genre-driven, and produced by speakers with a stable grammar. In that sense, the SLD comparison helps separate two questions that should not be collapsed: whether classifier-less Num + N strings are structurally interpretable in Mandarin at all, and why children sometimes produce them. The child corpus addresses the second question; headline-type SLD helps clarify the first.

This is where the comparison contributes something specific. In the SLD literature, headline innovation is treated as bounded innovation under special communicative pressure, not as free deletion: Xu and Qin (2015) identify headline language as one major SLD type, Dong and Huang (2019) argue that SLD innovations remain limited by broader grammatical architecture, and Yao and Xu (2021) relate headline innovation to information expansion and suppression rather than unconstrained rule breaking. Read in this light, the headline examples in the present article do not motivate the child trajectory and do not imply a shared derivational mechanism. Their contribution is narrower but still useful: they mark the adult edge of a bounded surface-compression space in Mandarin. This allows the child data to be interpreted more precisely—not as copies of headline grammar, but as developmentally distinct non-target forms whose surface shape falls within a type of compression that Mandarin can tolerate only under sharply delimited conditions. The comparison is therefore explanatory only in a weak clarificatory sense; its main value is descriptive and delimitative.

(16) 乃取一葫芦置于地

*Nai qu yi hulu zhi yu di*

Then take one gourd place at ground

“Then he took a gourd and placed it on the ground.” (Ouyang Xiu, *Zuiwengting ji*)

### 4.5 Limitations

The central limitation of the present study is data sparsity in the non-target domain. The developmental interpretation of omission rests on only four clear omission tokens in a corpus of 554 screened child records, and these four tokens are distributed across three children rather than concentrated in a single case. This distribution makes the phenomenon more than purely idiosyncratic, but it is still too sparse to support strong claims about a fully general developmental stage. The omission findings should therefore be read as cautious evidence of a patterned but rare developmental possibility.

The same caution applies to low-frequency classifier categories. The corpus does not show that standard or temporary classifiers are acquired only after age four, nor does it allow a direct causal inference from category rarity to cognitive load. The present findings are best treated as descriptive constraints on theory rather than as final adjudication among competing explanations.

Finally, corpora do not exhaust the child’s total input. The matched caregiver baseline captures distributional tendencies in recorded interaction, not the entirety of the child’s linguistic environment. Even so, corpus analysis remains valuable because it reveals what kinds of forms are actually available and recurrent in the child’s day-to-day interactional experience (Nazzal and Gavarró, 2025; Stärk et al., 2021).

## 5 Conclusion

Based on pre-screened *yi*-target records from CHILDES-TCCM (MacWhinney, 2000) and a matched caregiver-input baseline, this study offers a more transparent corpus analysis of early Mandarin numeral–classifier development while preserving the original comparison with Special Language Domain materials. The results show that overt *yi* + classifier forms overwhelmingly dominate the child data, that individual classifiers are even more strongly favored in child output than in caregiver input, and that clear omission is rare but structurally restricted. The general classifier ge functions partly as an early broad-use form, but only a small subset of *yi ge* + N tokens constitute clear overgeneralization.

Three cautiously stated claims follow from these findings. First, numeral–classifier development is staged rather than synchronously holistic. Second, the rare child errors observed here—only four clear omissions across three children—are structurally constrained and developmentally interpretable, not random strings. Third, the comparison with Special Language Domain data is best understood as a qualitative and descriptive one. Its value lies not in attributing a common derivation to child omissions and adult compressed genres, but in using adult headline material as a contrast case that helps delimit the surface compression Mandarin can sustain while remaining interpretable.

## Data Availability

The original contributions presented in the study are included in the article/supplementary material, further inquiries can be directed to the corresponding author/s.
